# Human umbilical cord mesenchymal stromal cells promotes the proliferation and osteogenic differentiation of autologous bone marrow stem cells by secreting exosomes

**DOI:** 10.1080/21655979.2022.2062183

**Published:** 2022-04-12

**Authors:** Yao Hai, Cao Zhidong, Wu Wenyan

**Affiliations:** a Department of Orthopedics, Chongqing Emergency Medical Center, Chongqing University Center Hospital, School of Medicine, Chongqing University, Chongqing, China; b Department of Clinical Laboratory, Chongqing Emergency Medical Center, Chongqing University Center Hospital, School of Medicine, Chongqing University, Chongqing, China

**Keywords:** hUMSC, exosomes, ABMSC, osteogenic differentiation

## Abstract

Fractures are frequently encountered diseases troubling the senior population, and the research on fracture repair and the exploration of effective treatment methods are of great significance. This study aimed to clarify the effect of human umbilical cord mesenchymal stromal cell-derived extracellular vesicles (hUMSC-EVs) on the proliferation and osteogenic differentiation of autologous bone marrow stem cells (ABMSCs). The two kinds of cells were co-cultured firstly, 5-Ethynyl-2’- deoxyuridine (EDU) staining and alizarin red staining were used to detect the proliferation and osteogenic differentiation of ABMSCs. The exosomes of hUMSCs were subsequently extracted to process ABMSCs to further test the effect on the cells. The EDU positive rate of ABMSCs and Collagen II expression were elevated, whereas the TdT-mediated dUTP nick end labeling (TUNEL) positive rate and Matrix Metallopeptidase 13 (MMP13) were markedly decreased after the co-culture of hUMSCs and ABMSCs using Transwell chamber assays. The results indicated that hUMSCs could increase the proliferation of ABMSCs, reduce apoptosis, and promote matrix metabolism. The hUMSCs exosomes were separated and added to ABMSCs. As the exosomes content increased, the proliferation of ABMSCs increased simultaneously, and ABMSCs apoptosis decreased. Meanwhile, ABMSCs that migrated to the submembrane increased compared with untreated ABMSCs. Western blot, qPCR and immunofluorescence results revealed that increased exosomes contents promoted the expression of ABMSCs anabolic-related indicators gradually, while decreased the expression of catabolism-related indicators gradually. The previously described results indicated that hUMSCs promoted the proliferation and osteogenic differentiation of ABMSCs by secreting exosomes.

## Graphical Abstract

**Figure:**
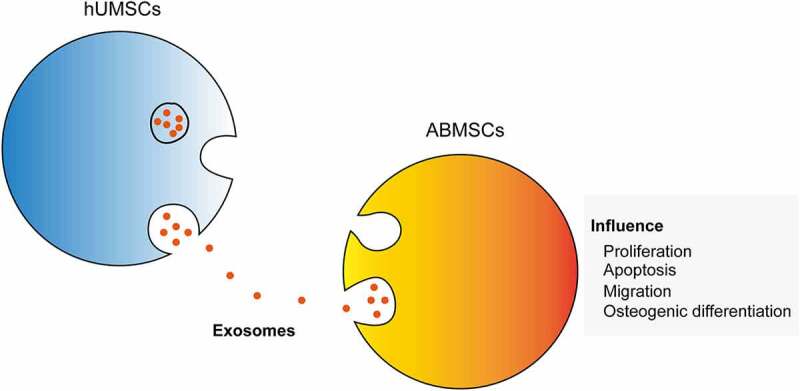

## 1 Introduction

Hip fractures are serious injuries that occur within 5 cm of the edge of the femoral head and the distal end of the lesser trochanter, and mainly include femoral neck fractures and intertrochanteric fractures [1]. The disease in seniors is more frequent. As the population in China gets older, the incidence of senior hip fractures is rising year by year. Decreased bone density is a recognized risk factor for hip fractures. Within 50–80 years of age, the risk of hip fractures increase by 4 times due to the decreased bone density [2]. Senior hip fracture victims, who have comorbidities in the circulatory, respiratory, digestive, neurological, and urinary systems after staying in bed, will soon develop lung and urinary system infections, deep vein thrombosis in the lower extremities, bedsores, cardiovascular and cerebrovascular accidents, and multiple systemic organ failure. Mortality during the perioperative period and one year after surgery is extremely high at 33%. Relative animal experiments have revealed that more new bone tissues are generated after mesenchymal stem cells implantation in the site of osteoporosis, trabecular density of the local new bone increases, and the bone tension test results are improved, which is helpful to increase the local bone density [3,4].

Exosomes are recognized as nanoscale membrane particles secreted by cells. They are transported into the environment by cells in the form of exocytosis. They function by fusion with target cells to release the active ingredients in them, and play a role in information exchange among cells [5,6]. Human umbilical cord mesenchymal stromal cells (hUMSCs) is a pluripotent stem cell and has advantages of high differentiation potential, easy acquisition and low immunogenicity. It has been widely introduced in the research of various tissue repair [7]. Some latest research findings have discovered that hUMSC-derived exosomes (hUMSC-EVs) can rejuvenate autologous bone marrow stem cells (ABMSCs) and effectively slow down the aging. In the context of treatment, the recovery effect of hUMSC-EVs on ABMSCs enhances the bone formation, wound healing and angiogenesis of bone tissues [8,9].

To alleviate the pain of senior hip fracture patients, the present study aimed to study the effect of hUMSC-EVs on the proliferation, apoptosis and osteogenic differentiation of ABMSCs. In addition, based on the existing research reports that hUMSCs promote the functional recovery of ABMSCs, enhance bone formation and wound healing, we speculated that hUMSC-EVs could effectively promote the proliferation and osteogenic differentiation of ABMSCs. Immunofluorescence, Western blot, and fluorescence quantitative PCR were performed to explore the effects of hUMSCs and different concentrations of hUMSC-EVs on proliferation, apoptosis, extracellular matrix metabolism and migration, and osteogenic differentiation of bone marrow mesenchymal stem cells at the cell level. The designed study was expected to provide the illustration of how hUMSC-EVs rejuvenate ABMSCs and effectively delay cells aging and a direction for treatment method.

## 2 Materials and methods

To investigate the efficacy of hUMSCs on the proliferation, and osteogenic differentiation of ABMSCs and clarify the hUMSC-EVs role in cell viability, proliferation, apoptosis, extracellular matrix metabolism and migration, and osteogenic differentiation of ABMSCs. Experiments in vitro were designed and carried out, immunofluorescence, Western blot, Edu staining, TUNEL assay, and fluorescence quantitative PCR were performed to validate the hypothesis that hUMSCs promote the proliferation, and osteogenic differentiation of ABMSCs through secreting exosomes.

### 2.1 Culture and passage of hUMSCs and ABMSCs

hUMSCs and ABMSCs applied in experiments were purchased from Chongqing Biomedicine Biotechnology Co., Ltd. The cells were cultured in dulbecco’s modified eagle medium (DMEM) containing 15% fetal bovine serum (FBS), in an incubator at 37°C, 5% CO_2_ and saturated humidity.

### 2.2 Induction and differentiation of hUMSCs and ABMSCs

#### Osteogenic differentiation

When MSCs fusion was at approximately 85%, the cells were digested using digestive solution and resuspended with hUMSCs complete medium. The cells at a density of 4 × 10^4^ cells/cm^2^ were inoculated into six-well plates pretreated with gelatin, and incubated with hUMSCs complete medium 2 mL per well in a 37°C, 5% CO_2_ incubator. When the cell fusion reached 60%, the culture supernatant was discarded. hUMSCs osteogenic differentiation complete medium was added and cultured in a 37°C, 5% CO_2_ incubator. hUMSCs osteogenic differentiation complete medium was replaced every 3 days, and the culture lasted for 2–4 weeks. When the induction culture stopped, the culture supernatant was discarded. The cells were subsequently washed twice with dulbecco’s phosphate-buffered Saline (DPBS), fixed with 4% neutral paraformaldehyde solution at room temperature for 20 min, stained with alizarin red staining solution for 15 min, observed and photographed under a microscope [10].

#### Adipogenic induction

When MSCs fusion was at approximately 85%, the cells were digested using digestive solution and resuspended with hUMSCs complete medium. The cells at a density of 4 × 104 cells/cm^2^ were inoculated into six-well plates pretreated with gelatin and incubated with hUMSCs complete medium 2 mL per well in a 37°C, 5% CO_2_ incubator. When the cell fusion reached 80%, the original culture supernatant was discarded. Induction solution A was supplemented for culture in a 37°C, 5% CO_2_ incubator. After 72 h of induction culture, the original culture supernatant was discarded, and induction solution B was added. After 24 h of induction culture, the original culture supernatant was discarded, and induction solution A was added and cultured in a 37°C, 5% CO_2_ incubator. The previously described procedures were repeated 3 times. When obvious lipid droplets appeared in the cells, the solution replaced with the induction solution B and continued the culture. The fresh induction solution B was replaced every 2 days, and continued the culture until the lipid droplets were large enough. When the induction culture stopped, the culture supernatant was discarded. The cells were fixed with 4% neutral paraformaldehyde solution at room temperature for 20 min, and stained by supplementing oil red O staining solution for 15 min, observed and photographed under a microscope [11].

#### Chondrogenic induction

When the MSCs were fused to approximate 85%, the cells are digested with digestion solution, and cell precipitate was resuspended with pretreated chondrogenic induction, centrifugated at 150 g for 5 min, resuspended using pretreated solution. When the cell density was about 1 × 10^6^ cells/ml, centrifugation was performed again and the supernatant was discarded. The precipitate was resuspended in chondrogenic induction complete medium, and the cell density was adjusted to 5 × 10^5^ cells/ml. Of 500 μl cell suspension was inoculated in a 15 ml centrifuge tube, centrifuged at 150 g for 5 min, and placed in a 37°C, 5% CO_2_ incubator for static culture. The complete induction medium was refreshed every 2 days. When the medium was changed, the used medium was gently aspirated, and added 500 μl of freshly prepared chondrogenic differentiation complete medium to each tube. After the induction culture, the cells were fixed with 4% neutral paraformaldehyde solution at room temperature for 1 h. Dehydration and paraffin embedding were conducted before section preparation. Following alcian blue staining, the cells were observed and photographed under a microscope [12].

### 2.3 Establishment of co-cultivation system

A transwell chamber at 0.4 μm pore diameter was applied to establish a co-cultivation system. The ABMSCs at 1 × 10^6^/well were inoculated in the lower chamber and hUMSCs at 5 × 10^5^/well in the upper chamber. The co-cultivation time lasted 72 h.

### 2.4 Detection of cell proliferation ability

#### EdU assay

ABMSCs were inoculated into 96-well plates at a density of 2 × 10^3^/well. The medium was DMEM/F12 medium free of exosomal serum, and different doses of exosomes (1, 5, and 10 mg) were added and co-cultured for 12 h. An equal volume of EdU working solution was added to the culture medium and co-cultured for 4 h. Subsequently, the cells were fixed with 4% paraformaldehyde and permeated with 0.1% Triton-100, followed by the addition of appropriate volume of the reaction solution [13]. Anti-quenching sealing agent containing DAPI were used to seal the film, which was observed and photographed using a fluorescence microscope.

### 2.5 Apoptosis detection

#### TUNEL detection

ABMSCs were inoculated into 96-well plates at a density of 2 × 10^3^/well. The medium was DMEM/F12 medium free of exosomal serum, and different doses of exosomes (1, 5, and 10 mg) were added and co-cultured for 12 h. The cells were fixed with 4% paraformaldehyde, transparent with 0.1% Triton-100, added with the TUNEL solution, and incubated for 1 h at 37°C in the dark. Anti-quenching sealing agent containing DAPI were used to seal the film, which was observed using a fluorescence microscope [14].

#### Annexin V/PI double staining flow cytometry

Co-culture with different doses of exosomes were conducted for 48 h. After digestion with ethylene diamine tetraacetic acid (EDTA)-free trypsin, the cells were collected, washed and resuspended using BB solution, 5 μl Annexin V and 5 μl propidium iodide (PI) were added to each test tube, and kept at room temperature in the dark for 15 min. The cells were washed and resuspended in the BB solution, and detected using flow cytometry within 30 min. The experimental data were analyzed via FlowJo^TM^V10 software.

### 2.6 Cell migration assay

Both inflammatory and normal chondrocytes were inoculated into the upper chamber of the transwell insert, and different doses of exosomes were supplemented to the medium in the lower chamber and incubated for 12 h. The cells were subsequently fixed in the upper chamber by adding with 4% paraformaldehyde, and the cells in the upper membrane that had not migrated were removed using a cotton swab. The cells in the lower layer were stained by adding with Giemsa staining solution [15]. The cells were ultimately photographed by randomly selecting five fields of each well, and counted using Image J software.

### 2.7 Immunofluorescence

The cells to be tested were fixed with 4% paraformaldehyde for 30 min, and washed twice with PBS. Of 0.5% Triton X-100 was added to permeate the membrane for 5 min, washed twice with PBS, added immunostaining blocking solution and blocked for 30 min. Primary antibodies were diluted at a ratio of 1:200, added an appropriate volume to the surface of the cell slide by dropwise, and co-cultured at 4°C for 16 h. Unbound primary antibodies were removed followed two cycles of washing with PBS, added with fluorescent-labeled secondary antibodies, incubated at 37°C for 1 h in the dark, and washed twice with PBS to remove unbound secondary antibodies. Anti-quenching sealing agent containing DAPI were used to seal the film, which was observed and photographed using a fluorescence microscope [16].

### 2.8 Extraction and identification of exosomes

The exosomes of bone marrow mesenchymal stem cells were extracted using the exosome extraction kit from Chongqing Biomedicine Biotechnology Co., Ltd. P2 generation ABMSCs were cultured in L-DMEM prepared from serum free of exosomes for 24 h, 20 ml of cell culture supernatant was collected, centrifuged at 3000 g for 15 min, the precipitate was discarded, and the supernatant extraction reagent was taken at a ratio of 5:1, let standby at 4°C for at least 12 h, and then placed in a 1500 g centrifugal machine for 35 min to obtain exosomes precipitation. The harvested precipitation was resuspended in PBS and pipetted repeatedly until the pellets were dissolved.

#### The obtained exosomes were identified using following detections

##### Nanometer tracking analysis (NTA) detection

The separated exosomes were diluted appropriately, uploaded to a Nano Sight nano particle size analyzer, and recorded the obtained particle concentration and particle size distribution of the samples [17].

##### Western blot detection

The exosomal samples were performed protein quantification, directly added an appropriate amount of protein loading buffer, and denatured by boiling [18]. The hUMSCs protein extract was used as a control group to detect glucose-regulated protein 94 (GRP94), tumor susceptibility gene 101 (TSG101), heat shock protein 70 (HSP70), CD9, CD63, and CD81. The antibodies were purchased from Abcam (Abcam Trading (Shanghai) Co., Ltd.).

### 2.9 CCK8 assay

ABMSCs were inoculated into 96-well plates at a density of 2 × 10^3^/well. The medium was DMEM/F12 medium free of exosomal serum, and different doses of exosomes (1, 5, and 10 mg) were added. The medium was replaced every day and added with CCK-8 reagent. Following 2 h, a microplate reader was employed to measure each well at 450 nm and 620 nm wavelengths, and the cell proliferation curve of each group was plotted.

### 2.10 Detection of the ABMSCs ability of exosome uptake

Exosome fluorescent labeling: the exosome samples were added with 200 μl diluent C and mixed well. Meanwhile, 300 μl of diluent solution C and 4 μl of PKH67 were added to a sterile EP tube and mixed well. The previously described two tubes were mixed well and incubated subsequently at room temperature for 5 min. Of 600 μl 1% BSA was supplied to stop staining for 1 min, and the mixture was added with 1% BSA to make a volume of 20 ml. Ultracentrifugation at 120,000 g was performed for 60 min. The supernatant was discarded, and PKH67-exosomal precipitation was resuspended in sterile PBS.

#### Co-incubation

The cell culture supernatant was discarded. The cells were rinsed with PBS three times, and added with the complete medium containing exosome-free serum. The freshly prepared PKH67-exosomes were added to the cells to make the final concentration at 10 μg/ml. The control group used an equal amount of staining solution and added to sterile PBS, and incubated with the cells. After being mixed well, the cells were incubated in a 37°C, 5% CO_2_ cell incubator for 12 h.

#### Tracing

After the incubation completed, the cells were washed twice with pre-warmed PBS, fixed with 4% paraformaldehyde at room temperature for 10 min, washed twice with PBS, added with 0.1% Triton X-100 for treatment for 5 min, and washed twice with PBS. Rhodamine phalloidin was diluted at a ratio of 1:800, added 200 μl to each slide, incubated at room temperature for 30 min in the dark, and washed twice with PBS. A sealing agent containing DAPI were used to seal the slide, which was observed and photographed using a laser confocal microscope.

### 2.11 Western blotting

The cells were lysed on ice using a RIPA lysis buffer which was mixed with protease inhibitors and phosphatase inhibitors. After 5 min, the cell lysates and cell debris were collected in an EP tube using a cell scraper, and centrifuged at 12,000 g for 20 min. The supernatant was collected to determine protein concentration, added with a loading buffer, quantified the protein concentration to 1 ng/μl, and boiled at 100 degrees for 10 min. Ten microliter protein samples were added to each SDS-PAGE gel well. The sample was placed in the concentrated gel, electrophoresis was carried out at a constant voltage of 80 V. After the sample entered the separation gel, the voltage was adjusted to 100 V until the bromophenol blue indicator band in the samples reached the bottom of the gel and the electrophoresis was terminated. A polyvinylidene fluoride (PVDF) membrane was trimmed equivalent to the size of the gel, covered on the gel after methanol activation, and added with pre-cooled transfer fluid to membrane transfer. Under a low-temperature environment, the membrane was transferred at a constant current of 200 mA for 90 min. After the transfer, the PVDF membrane was placed into an incubator containing fast blocking solution, and sealed at 37°C for 15 min. Primary antibody diluent was applied to dilute primary antibodies at 1:1000 ratio, placed into a hybridization incubation bag, and incubated the sealed PVDF membrane with the primary antibody for 16 h at 4°C. The PVDF membrane incubated with primary antibody was washed using PBS with 0.1% Tween 20 (PBST) 4 times, 10 min each time. Secondary antibodies were diluted at 1:10,000 ratio, and incubated with the PVDF membrane at 37°C for 1 h. An appropriate amount of ECL chemiluminescent liquid was dripped on the PVDF membrane, and exposed using a gel imager in a low-temperature environment away from light. The experimental results were analyzed using Image J software.

### 2.12 Real-time fluorescence quantitative PCR

#### RNA extraction

After the medium was aspirated, the cell surface was washed with PBS, supplemented with an appropriate amount of TRIzol, and lysed at room temperature for 10 min. Chloroform at one-fifth of the lysate volume was added, mixed well by vibrating for 30s, and let stand for 5 min at room temperature. The mixture was centrifuged at 12,000 g for 20 min at 4°C until obvious liquid stratification was observed in the EP tube. The upper transparent liquid was transferred to an enzyme-free EP tube using an RNase-free tip, added with an equal volume of isopropanol to the supernatant, mixed well by up-down reversal, and let stand at room temperature for 10 min. The solution was centrifuged at 12,000 g for 20 min in a 4°C environment until white RNA precipitates at the bottom of the EP tube was visible. To remove the organic solvent, an appropriate amount of 75% ethanol was added to wash the precipitate twice. Alcohol residue was carefully removed. When the precipitate was slightly dry, a small amount of diethylpyrocarbonate (DEPC)-treated water was added to dissolve the precipitate.

#### Reverse transcription

RNA concentration of the extracted samples was determined, processed according to the instructions of the reverse transcription kit and the genomic DNA in the samples was removed, and then reverse transcribed the RNA into cDNA, and the samples were stored at −20°C.

#### Real-time fluorescence quantitative PCR

The target primers were designed first, and they were synthesized by Chongqing Biomedicine Biotechnology Co., Ltd. The procedures were performed according to the kit instructions under a dark environment. Samples, primers and amplifying enzyme mixture were added, and three multiple holes were set for each index in each group. After verifying the amplification efficiency of the primers, real-time fluorescent quantitative PCR detection was conducted. The experimental results were evaluated using Step one 2.0 software on amplification curve, dissolution curve, and multiple hole differences of the experimental results. The data obtained were exported, and the CT value obtained in the experiment was processed using a 2^−ΔΔCT^ method. GraphPad Prism 8.0 software was employed for data analysis and graphing.

### 2.13 Statistical analysis

At least three biological replicates were performed for each experiment. All data were expressed as mean ± standard deviation (SD), and data analysis and graphical display were performed by GraphPad Prism 8.0 software. Independent sample t tests were conducted to compare the difference between two groups, and one-way analysis of variance was used to compare the difference between multiple groups. P < 0.05 was considered statistically significant.

## 3. Results

### 3.1 Identification of hUMSCs and ABMSCs

**Figure 1.**
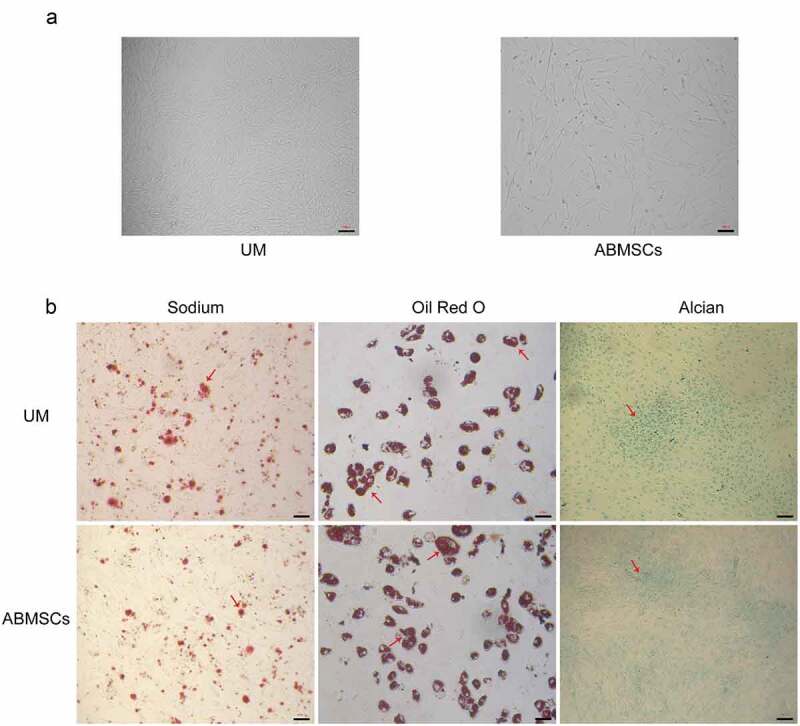
Culture and identification of hUMSCs and ABMSCs. (a) Fusiform morphology of two mesenchymal stem cells was observed under a light microscope. (b) MSCs were induced in osteogenic, chondrogenic and lipogenic media for 4 weeks and were subsequently stained with alizarin red (left), oil red O (middle) and Alcian blue (right), respectively. Scale bar is 100 µm.

At the 4th passage, both MSCs presented a typical spindle-shaped morphology (Figure 1a). MSCs were induced in osteogenic, chondrogenic and lipogenic media for 4 weeks. Osteogenesis-induced MSCs were stained using alizarin red, and stained calcium mineral deposits were visible (Figure 1b, left) to determine the osteogenic ability. Lipogenesis-induced MSCs were stained with oil red O with visible stained lipid droplets (Figure 1b, middle) to determine the adipogenic ability. Cartilage globules were formed by chondrogenesis-induced MSCs. The cartilage globules were cut into thin slices and stained with alcian blue. The stained acid mucopolysaccharide (Figure 1b, right) was seen to determine the chondrogenesis ability.

### 3.2 hUMSCs can improve the metabolism of ABMSCs

The co-culture tests were performed to detect the regulatory effect of hUMSCs on ABMSCs. The hUMSCs cultured in the upper layer could not directly pass through the cellulose membrane with a diameter of 0.4 μm, but the secreted substances could pass through this membrane and affected the metabolism of the ABMSCs cultured in lower layer. As per whether they were co-cultured with hUMSCs, ABMSCs were divided into two groups: one group of ABMSCs was cultured alone and the other was co-cultured with hUMSCs. Cell apoptosis, proliferation, migration, and extracellular matrix were determined using immunofluorescence and cell migration experiments.

**Figure 2.**
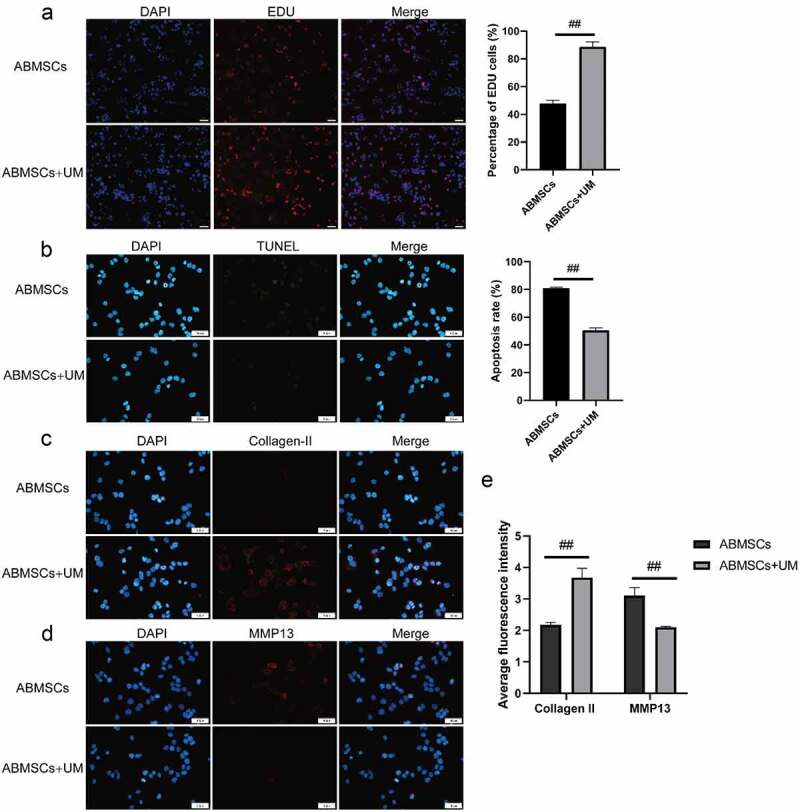
hUMSCs can improve ABMSCs metabolism. (a) EdU assay was performed to detect cell proliferation. (b) Detection of cell apoptosis by TUNEL. (c) Detection of Collagen II by immunofluorescence assay. (d) Detection of MMP13 by immunofluorescence assay. (e) Statistical results of the average fluorescence intensity. Scale bar is 50 µm.

The EDU tests were performed to detect cell proliferation. As shown in Figure 2a, the positive rate of ABMSCs co-cultured with hUMSCs was higher than that of ABMSCs cultured alone. The results showed that hUMSCs could improve the proliferation ability of normal ABMSCs through paracrine. The fluorescence TUNEL tests were conducted to detect ABMSC apoptosis after co-culture. As shown in Figure 2b, the positive rate of TUNEL in ABMSCs co-cultured with hUMSCs was markedly reduced. The previously obtained results indicated that hUMSCs could inhibit ABMSCs apoptosis.

We subsequently detect the metabolism changes in extracellular matrix. Collagen II was selected as a relevant indicator of ABMSCs extramatrix anabolism, and MMP13 as a relevant indicator of ABMSCs extramatrix catabolism. The results revealed that ABMSCs co-cultured with hUMSCs increased markedly in Collagen II expression (Figure 2c) but decreased greatly in MMP13 (Figure 2d) compared with ABMSCs cultured alone. The findings indicated that hUMSCs could improve the matrix metabolism of ABMSCs (Figure 2e).

### 3.3 Identification of exosomes

**Figure 3.**
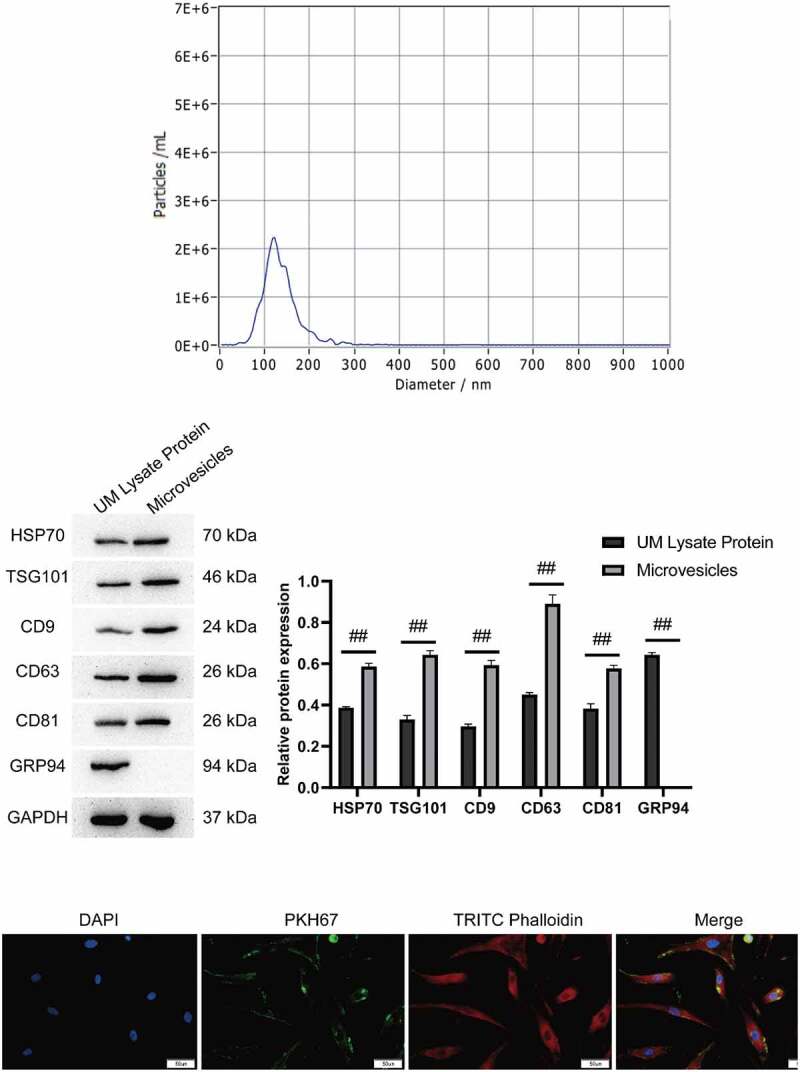
Identification of hUMSC-EVs. A, Detection of exosome particle diameters by NTA. B, Western blot was used to detect exosome markers HSP70, TSG101, CD9, CD63 and CD81. C, PKH67 labeled exosomes were co-cultured with ABMSCs for 12 h, and the green fluorescent-labeled exosomes entering the ABMSCs skeleton range labeled by red fluorescence were observed under a confocal microscope. Scale bar is 50 µm.

We believed that hUMSCs regulated the activity of ABMSCs by secreting exosomes. Exosomes extracted from the supernatant of hUMSCs medium were identified using NTA and Western blotting assays. NTA detection revealed that the diameter of particles in exosomal suspension following separation, extraction and purification was 103.8 ± 31.6 nm (accounting for 98.83 ± 0.38%), which was basically consistent with the results of TEM. The concentration of the isolated and extracted exosomes was about 3.2 × 10^10^/ml (Figure 3a). Western blotting technology was employed to detect the common characteristic surface markers of exosomes in the suspension of exosomes to be identified. Following electrophoresis, Western blotting and enzyme-linked immunoassay, the specific proteins in the suspension were screened out. Based on final Western blotting results, the exosomal markers HSP70, TSG101, CD9, CD63 and CD81 were highly expressed in the exosomes which was higher than that in hUMSCs, the endoplasmic reticulum marker GRP94 was not expressed in the exosomes extracted, whereas it were highly expressed in hUMSCs (Figure 3b). The above results indicated that the extracted exosomes met the identification criteria for exosomes.

To confirm that the exosomes secreted by hUMSCs was available for takenup by ABMSCs, this study co-cultured PKH67-labeled exosomes with ABMSCs for 12 h. As shown in Figure 3c, the green fluorescence labeled exosomes were visualized to enter the skeleton range of red fluorescence labeled ABMSCs and were evenly distributed in the cytoplasm under a confocal microscope.

### 3.4 Exosomes can improve the metabolism of ABMSCs

To verify that exosomes altered the metabolism of ABMSCs, we applied gradient doses (1 × 10^10^, 5 × 10^10^, and 10 × 10^10^/ml exosomes) to intervene ABMSCs to observe the proliferation, apoptosis, extracellular matrix metabolism, migration and osteogenic differentiation of ABMSCs. ABMSCs were divided into the following four groups: (1) Untreated ABMSCs, (2) ABMSCs + 1 × 10^10^ exosomes, (3) ABMSCs + 5 × 10^10^ exosomes, and (4) ABMSCs + 10 × 10^10^ exosomes.

**Figure 4.**
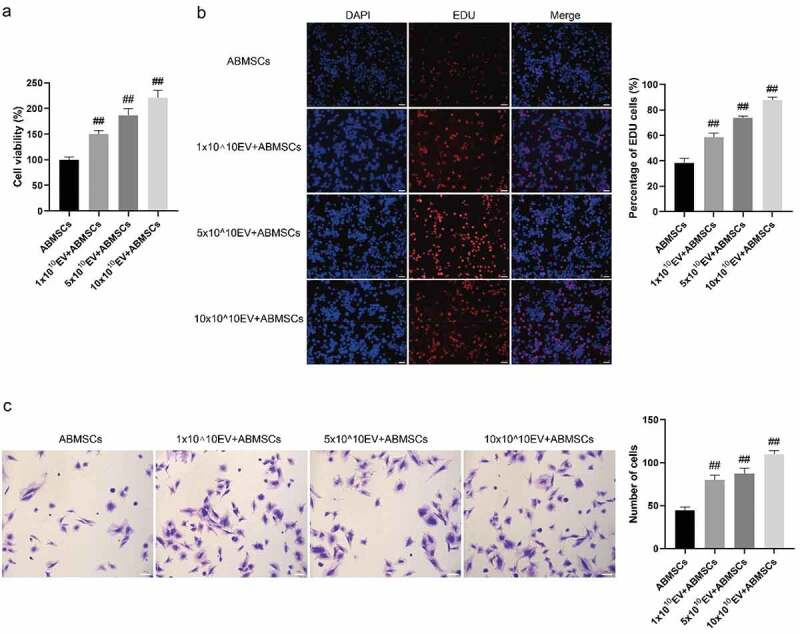
hUMSC-EVs promotes ABMSCs proliferation and migration. (a) CELL proliferation of untreated ABMSCs, ABMSCs + 1 × 10^10^ exosomes, ABMSCs + 5 × 10^10^ exosomes, and ABMSCs + 10 × 10^10^ exosomes was detected by CCK8 method. (b) EdU staining to detect cell proliferation. Scale bar is 50 µm. (c) Detection of cell migration by Transwell. Scale bar is 100 µm.

CCK8 proliferation detection and EDU fluorescence staining method were applied to evaluate the proliferation phenotype of ABMSCs. As shown in Figure 4a, the cell proliferation curve indicated that as the amount of exosomes increased, the proliferation rate of ABMSCs also increased markedly. Positive EDU staining indicated that the cells were in the proliferation stage. As shown in Figure 4b, compared with untreated ABMSCs, as the amount of exosomes increased, the positive rate of EDU staining for ABMSCs also increased substantially. Transwell chamber was applied to evaluate the changes in the migration phenotype of untreated ABMSCs. As shown in Figure 4c, as the amount of exosomes increased, ABMSCs migrating to the submembrane increased more than untreated ABMSCs.

**Figure 5.**
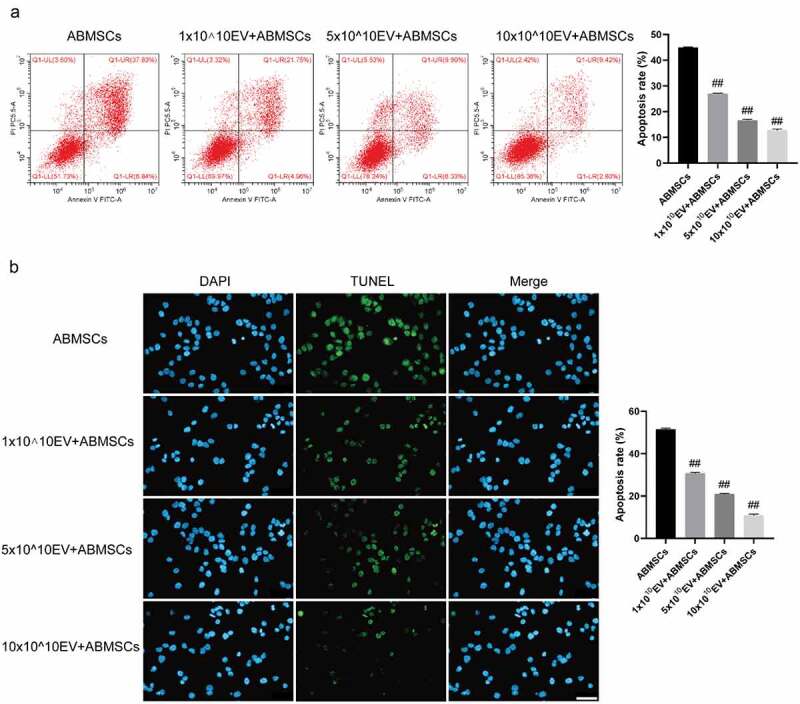
UMSC-EVs inhibits apoptosis of ABMSCs. (a) Detection of cell apoptosis by flow cytometry. (b) Detection of cell apoptosis by TUNEL. Scale bar is 50 µm for all panels.

The Annexin V/PI double staining flow cytometry combined with the TUNEL fluorescent staining method was applied to detect apoptosis of ABMSCs to evaluate the changes in the apoptotic phenotype of ABMSCs. As shown in Figures 5A and 5b, as the amount of exosomes increased, the ratio of apoptotic cells in ABMSCs decreased. Positive TUNEL staining indicated that the cells were in the apoptotic stage. As the amount of exosomes increased, the positive rate of TUNEL staining for ABMSCs was lower than that of untreated ABMSCs.

**Figure 6.**
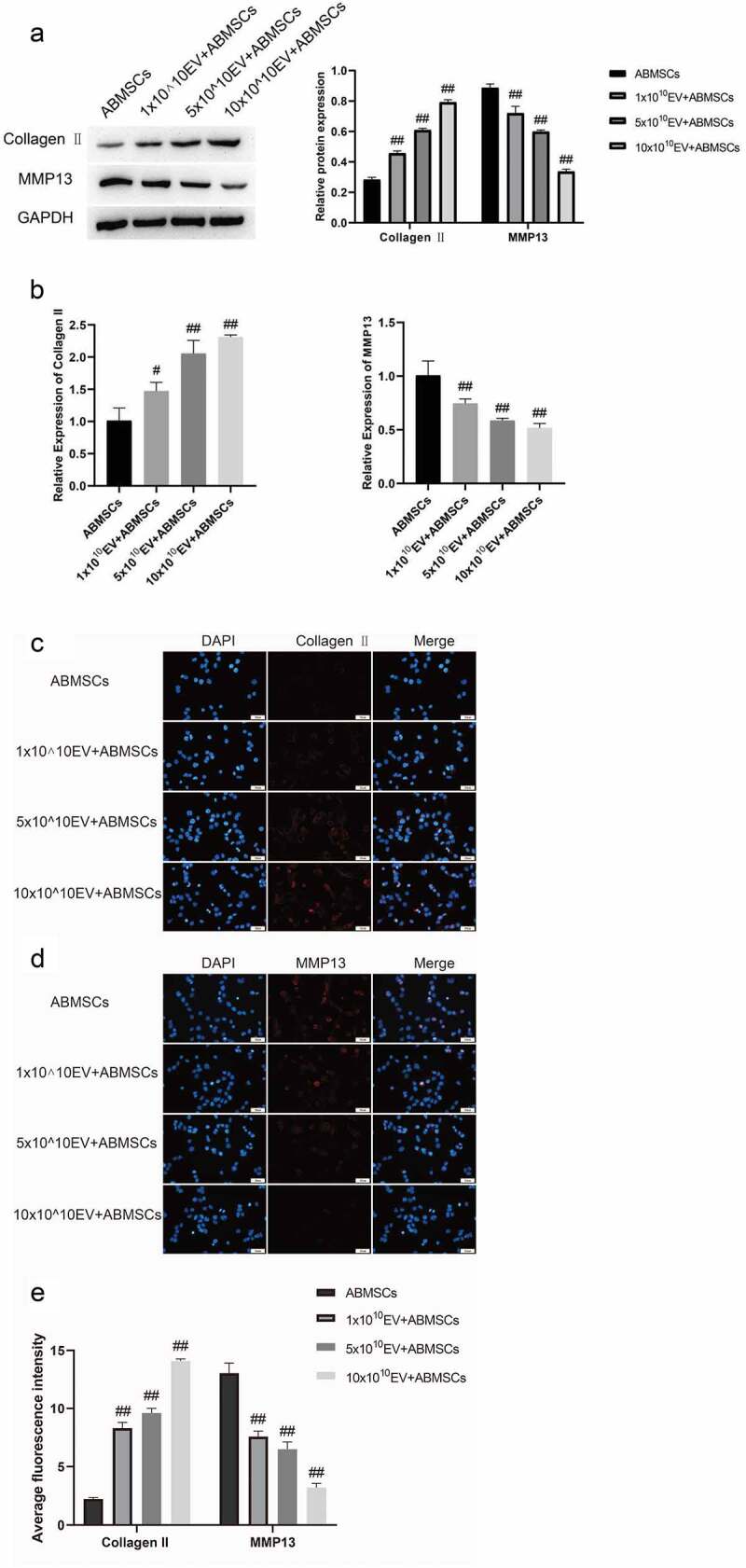
UMSC-EVs promotes Collagen II expression and inhibits MMP13 expression. (a) Detection of Collagen II and MMP13 expressions by Western blotting. (b) Detection of Collagen II and MMP13 expressions by qPCR. (c-e) Detection of Collagen II and MMP13 expressions by immunofluorescence assay.

Collagen II was selected as a relevant indicator of ABMSCs extramatrix anabolism, and MMP13 as a relevant indicator of ABMSCs extramatrix catabolism. Western blotting assays were conducted to determine the changes in protein extracellular matrix-related metabolic indicators at the protein level. Compared with untreated ABMSCs, the addition of exosomal ABMSCs increased the expression of anabolic-related indicators as the amount of exosomes increased, while catabolic related indicators gradually decreased (Figure 6a). Real-time fluorescent quantitative PCR was used to detect changes in chondrocytes at the RNA level. As the amount of exosomes increased, the expression of ABMSCs anabolic-related indicators gradually increased, while the expression of catabolism-related indicators gradually decreased (Figure 6b). The immunofluorescence results (Figure 6c-e) indicated that as the amount of exosomes increased, the fluorescence intensity of MMP13 gradually decreased, and the fluorescence intensity of Collagen II increased gradually. The above-mentioned results revealed that, compared with untreated ABMSCs, with the increase of the dose of exosomes, the proliferation, migration, apoptosis, and exo-matrix metabolism capabilities of ABMSCs were markedly improved.

## 4 Discussion

In the past few decades, MSC-based therapies have been shown to trigger complex interactions between multiple types of cells, extracellular matrix components, and post-damage signaling molecules. It has been reported that this type of therapy can promote bone regeneration and fracture healing [19,20]. Among several potential molecular mechanisms, the exosomes released by MSCs play a crucial role and have attracted increasing concerns. As the number, proliferation and differentiation potential of adult hUMSCs gradually decrease, there is a higher rate of viral infection, and the derivation is easily restricted. The further research on MSCs continues, it is feasible to isolate MSCs from umbilical cord blood, umbilical cord, lipid, skeletal muscle cells, and the liver tissues [21,22]. Compared with other sources of MSCs, hUMSCs originated MSCs has advantages of extensive origin and easy separation and it is not subject to ethical restrictions [23,24].

The process of bone formation is a process of balancing the activity of osteoblasts and osteoclasts in the bones. The role of osteoblasts is to synthesize bone matrix, which is differentiated from ABMSCs [25,26]. The process of fracture healing requires the production of a large number of new osteoblasts, which accelerates bone synthesis and calcification, and increases bone volume and density [27]. As the body ages, the number of ABMSCs functional cells decreases, and their potential to differentiate into osteoblasts in the bone marrow is down-regulated [28,29]. Meanwhile, senescence-related secretory phenotypic molecules act in the bone microenvironment and inhibit the osteogenic differentiation of ABMSCs.

The present study conducted flow cytometry and multi-line differentiation induction to verify the purity and multi-line differentiation potential of hUMSCs. A co-culture system of hUMSCs and ABMSCs was constructed for detection via immunofluorescence, Western blotting and real-time fluorescence quantitative PCR assays. The results demonstrated that hUMSCs had a positive effect on the proliferation, apoptosis, migration and extracellular matrix metabolism of ABMSCs. The proliferation rate was markedly increased, apoptosis was greatly reduced, the migration ability was substantially enhanced, and the extracellular matrix metabolism was largely improved. The addition of different concentrations of hUMSC-EVs could produce similar results to the co-culture with hUMSCs. Additionally, with the increase of the concentration of hUMSC-EVs added, the osteogenic differentiation of ABMSCs was markedly enhanced. It suggested that hUMSC-EVs could rejuvenate ABMSCs and effectively slow down the process of senescence.

As the aging population in China increases, the incidence of hip fractures in the elderly increases accordingly. The present treatment methods of hip fractures mainly include surgical and non-surgical options [30]. The frequently introduced surgical methods are artificial hemiarthroplasty also known as artificial femoral head replacement, artificial total hip replacement, closed reduction and internal fixation of femoral neck fractures with hollow lag screws, and proximal femoral nail antirotation [30]. The damaged parts of the hip joint, such as the femoral head and neck or hip acetabulum were replaced with artificial appropriate bone cemented or biological bipolar femoral head prosthesis by surgical operation [31,32]. Because senior victims of hip fractures develop multiple chronic diseases plus the weakened body organ functions, they are prone to various perioperative complications, which increase the surgical risks. Even if the perioperative period is safely passed, only some patients succeed in recovering to the pre-operative functions [33,34]. Our current research provides effective evidence for the first time to prove that exogenous stem cells and host endogenous progenitor cells make communications through intercellular exosomal signals, which improves cell proliferation and osteogenic differentiation in vitro, and this information may help to develop a new acellular bone healing therapy in the future.

## 5. Conclusions

hUMSCs promoted the proliferation and osteogenic differentiation of ABMSC by secreting exosomes. After hUMSC-EVs treatment, the EDU positive rate of ABMSCs and Collagen II expression were markedly elevated, whereas the TUNEL positive rate and MMP13 were markedly decreased.

## Highlights

1. hUMSCs can significantly inhibit ABMSCs apoptosis.
2. hUMSCs elevate Collagen II expression whereas decrease MMP13 expression.
3. hUMSCs improve the metabolism and migration of ABMSCs.
4. hUMSCs promote proliferation and osteogenic differentiation of ABMSCs.

## References

[1] Wong KC, Cheok JWG, Tay KXK, et al. Where have all the hip fractures gone? [J]. Osteoporos Int. 2020;31(10):2057–2058. 32488291 10.1007/s00198-020-05483-0 PMC7266647

[2] Hadjimichael AC. Hip fractures in the elderly without osteoporosis [J]. J Frailty Sarcopenia Falls. 2018;3(1):8–12. 32300689 10.22540/JFSF-03-008 PMC7155350

[3] Barrett-Lee J, Barbur S, Johns J, et al. Hip fractures in centenarians: a multicentre review of outcomes [J]. Ann R Coll Surg Engl. 2021;103(1):59–63. 32969242 10.1308/rcsann.2020.0203 PMC7705146

[4] Chinoy MA, Javed S. Frequency of vertebral fractures in patients presenting with hip fractures [J]. Pak J Med Sci. 2020;36(1):S44–s48. 31933606 10.12669/pjms.36.ICON-Suppl.1709 PMC6943115

[5] Ha DH, Kim HK, Lee J, et al. Mesenchymal stem/stromal cell-derived exosomes for immunomodulatory therapeutics and skin regeneration [J]. Cells. 2020;9:5. 10.3390/cells9051157 PMC7290908 32392899

[6] Liu W, Yu M, Xie D, et al. Melatonin-stimulated MSC-derived exosomes improve diabetic wound healing through regulating macrophage M1 and M2 polarization by targeting the PTEN/AKT pathway [J]. Stem Cell Res Ther. 2020;11(1):259. 32600435 10.1186/s13287-020-01756-x PMC7322868

[7] Sun D, Jiang Z, Chen Y, et al. MiR-455-5p upregulation in umbilical cord mesenchymal stem cells attenuates endometrial injury and promotes repair of damaged endometrium via Janus kinase/signal transducer and activator of transcription 3 signaling [J]. Bioengineered. 2021;12(2):12891–12904. 34784837 10.1080/21655979.2021.2006976 PMC8810187

[8] Nakao Y, Fukuda T, Zhang Q, et al. Exosomes from TNF-α-treated human gingiva-derived MSCs enhance M2 macrophage polarization and inhibit periodontal bone loss [J]. Acta Biomater. 2021;122:306–324. 33359765 10.1016/j.actbio.2020.12.046 PMC7897289

[9] Zuo R, Liu M, Wang Y, et al. BM-MSC-derived exosomes alleviate radiation-induced bone loss by restoring the function of recipient BM-MSCs and activating Wnt/β-catenin signaling [J]. Stem Cell Res Ther. 2019;10(1):30. 30646958 10.1186/s13287-018-1121-9 PMC6334443

[10] Fei D, Xia Y, Zhai Q, et al. Exosomes regulate interclonal communication on osteogenic differentiation among heterogeneous osteogenic single-cell clones through PINK1/Parkin-mediated mitophagy [J]. Front Cell Dev Biol. 2021;9:687258. 34604210 10.3389/fcell.2021.687258 PMC8484762

[11] Tooi M, Komaki M, Morioka C, et al. Placenta mesenchymal stem cell derived exosomes confer plasticity on fibroblasts [J]. J Cell Biochem. 2016;117(7):1658–1670. 26640165 10.1002/jcb.25459

[12] Weissenberger M, Weissenberger MH, Gilbert F, et al. Reduced hypertrophy in vitro after chondrogenic differentiation of adult human mesenchymal stem cells following adenoviral SOX9 gene delivery [J]. BMC Musculoskelet Disord. 2020;21(1):109. 32066427 10.1186/s12891-020-3137-4 PMC7026978

[13] Zeng C, Pan F, Jones LA, et al. Evaluation of 5-ethynyl-2’-deoxyuridine staining as a sensitive and reliable method for studying cell proliferation in the adult nervous system [J]. Brain Res. 2010;1319:21–32. 20064490 10.1016/j.brainres.2009.12.092 PMC2826567

[14] Le TM, Morimoto N, Ly NTM, et al. Ex vivo induction of apoptotic mesenchymal stem cell by high hydrostatic pressure [J]. Stem Cell Rev Rep. 2021;17(2):662–672. 33128169 10.1007/s12015-020-10071-0 PMC8036216

[15] Kramer N, Walzl A, Unger C, et al. In vitro cell migration and invasion assays [J]. Mutat Res. 2013;752(1):10–24. 22940039 10.1016/j.mrrev.2012.08.001

[16] Hua WB, Wu XH, Zhang YK, et al. Dysregulated miR-127-5p contributes to type II collagen degradation by targeting matrix metalloproteinase-13 in human intervertebral disc degeneration [J]. Biochimie. 2017;139:74–80. 28559201 10.1016/j.biochi.2017.05.018

[17] Street JM, Koritzinsky EH, Glispie DM, et al. Urine exosome isolation and characterization [J]. Methods Mol Biol. 2017;1641:413–423. 28748478 10.1007/978-1-4939-7172-5_23 PMC9374111

[18] Parvanian S, Zha H, Su D, et al. Exosomal vimentin from adipocyte progenitors protects fibroblasts against osmotic stress and inhibits apoptosis to enhance wound healing [J]. Int J Mol Sci. 2021;22:9. 10.3390/ijms22094678 PMC8125065 33925176

[19] Shao M, Xu Q, Wu Z, et al. Exosomes derived from human umbilical cord mesenchymal stem cells ameliorate IL-6-induced acute liver injury through miR-455-3p [J]. Stem Cell Res Ther. 2020;11(1):37. 31973730 10.1186/s13287-020-1550-0 PMC6979401

[20] Thomi G, Surbek D, Haesler V, et al. Exosomes derived from umbilical cord mesenchymal stem cells reduce microglia-mediated neuroinflammation in perinatal brain injury [J]. Stem Cell Res Ther. 2019;10(1):105. 30898154 10.1186/s13287-019-1207-z PMC6429800

[21] Yan L, Liu G, Wu X. Exosomes derived from umbilical cord mesenchymal stem cells in mechanical environment show improved osteochondral activity via upregulation of LncRNA H19 [J]. J Orthop Translat. 2021;26:111–120. 33437630 10.1016/j.jot.2020.03.005 PMC7773952

[22] Zhang L, Song Y, Chen L, et al. MiR-20a-containing exosomes from umbilical cord mesenchymal stem cells alleviates liver ischemia/reperfusion injury [J]. J Cell Physiol. 2020;235(4):3698–3710. 31566731 10.1002/jcp.29264 PMC13482650

[23] Atluri S, Manchikanti L, Hirsch JA. Expanded umbilical cord mesenchymal stem cells (UC-MSCs) as a therapeutic strategy in managing critically Ill COVID-19 patients: the case for compassionate use [J]. Pain Physician. 2020;23(2):E71–e83. 32214286

[24] Shang Y, Guan H, Zhou F. Biological characteristics of umbilical cord mesenchymal stem cells and its therapeutic potential for hematological disorders [J]. Front Cell Dev Biol. 2021;9:570179. 34012958 10.3389/fcell.2021.570179 PMC8126649

[25] Qin Y, Wang L, Gao Z, et al. Bone marrow stromal/stem cell-derived extracellular vesicles regulate osteoblast activity and differentiation in vitro and promote bone regeneration in vivo [J]. Sci Rep. 2016;6:21961. 26911789 10.1038/srep21961 PMC4766421

[26] Liao W, Ning Y, Xu H-J, et al. BMSC-derived exosomes carrying microRNA-122-5p promote proliferation of osteoblasts in osteonecrosis of the femoral head [J]. Clin Sci (Lond). 2019;133(18):1955–1975. 31387936 10.1042/CS20181064

[27] Wang B, Wu W, Xu K, et al. MicroRNA-223-3p is involved in fracture healing by regulating fibroblast growth factor receptor 2 [J]. Bioengineered. 2021;12(2):12040–12048. 34753389 10.1080/21655979.2021.2002498 PMC8810112

[28] Zhu F, Wang J, Ni Y, et al. Curculigoside protects against titanium particle-induced osteolysis through the enhancement of osteoblast differentiation and reduction of osteoclast formation [J]. J Immunol Res. 2021;2021:5707242. 34285923 10.1155/2021/5707242 PMC8275416

[29] Zheng J, Zhang X, Zhang Y, et al. Osteoblast differentiation of bone marrow stromal cells by femtosecond laser bone ablation [J]. Biomed Opt Express. 2020;11(2):885–894. 32206397 10.1364/BOE.383721 PMC7041461

[30] Parker MJ, Johansen A, Griffiths R. Bone cement and hip fractures [J]. Injury. 2021;52(7):1655–1656. 34030862 10.1016/j.injury.2021.05.004

[31] Szczesiul J, Bielecki M. A review of total hip arthroplasty comparison in FNF and OA patients [J]. Adv Orthop. 2021;2021:5563500. 34567807 10.1155/2021/5563500 PMC8463253

[32] Dabees S, Kamel BM, Tirth V, et al. Experimental design of Al2O3/MWCNT/HDPE hybrid nanocomposites for hip joint replacement [J]. Bioengineered. 2020;11(1):679–692. 32543986 10.1080/21655979.2020.1775943 PMC8291848

[33] Barry ML, Maday KR. Reviewing acute hip fractures in adults [J]. Jaapa. 2021;34(9):1–10. 10.1097/01.JAA.0000742968.43332.6e 34448784

[34] Goel A, Adhiyaman V, Chattopadhyay I. Have we improved in diagnosing hip fractures? [J]. J R Coll Physicians Edinb. 2020;50(2):207–214. 10.4997/JRCPE.2020.229 32568300

